# Blood pressure after follow‐up in a stroke prevention clinic

**DOI:** 10.1002/brb3.1667

**Published:** 2020-06-12

**Authors:** Agnete Hviid Hornnes, Mai Bang Poulsen

**Affiliations:** ^1^ Department of Neurology Herlev og Gentofte Hospital Herlev Denmark; ^2^ Department of Neurology Rigshospitalet Copenhagen Denmark

**Keywords:** blood pressure, blood pressure target, randomized controlled trial, secondary prevention, stroke recurrence

## Abstract

**Objectives:**

In Denmark, 25% of hospital admissions with stroke are recurrent strokes. With thrombolytic treatment, more patients survive with only minor disability. This promising development should be followed up by intensive secondary prevention. Hypertension is the most important target. We aimed at testing the hypotheses that early follow‐up in a preventive clinic would result in (a) a higher proportion of patients with blood pressure at target and (b) time to stroke recurrence, myocardial infarction, and death would be longer in the intervention group compared to controls.

**Materials and Methods:**

Eligible patients admitted to the stroke unit of Herlev Hospital were randomized shortly before discharge to intervention or control group. Of 78 included participants, data from 73 were available for follow‐up 9 months after inclusion. Patients in the intervention group were seen in the clinic within 1 week. In case of hypertension, treatment was initiated or supplied with a new drug. We used individual targets for blood pressure according to diagnosis of stroke and patients' comorbidity. Patients in the intervention group had a median of five visits to the preventive clinic.

**Results:**

In the intervention group, blood pressure was treated to target in 25 patients (69%) versus 14 (38%) in the control group (*p* = .007). Median time to first event was 44 months (4–49) in the intervention group and 19 months (4–37) in controls (*p* = .316).

**Conclusions:**

Treatment of hypertension to individual targets after stroke is feasible. It may postpone recurrent stroke and death in stroke survivors.

## Graphical abstract

In this randomized controlled trial, we showed that blood pressure can be treated to target in stroke survivors. Data indicate that this preventive effort may postpone recurrent stroke.

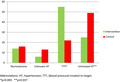

## 1 INTRODUCTION

Over the last two decades, continuous development of thrombolytic treatment of acute ischemic stroke (IS) has improved safety and functional outcome in treated patients (Wahlgren, 2009; Wahlgren et al., 2008) thus increasing the possibility of survival with no or only minor disability. With this fact and the ongoing aging of populations in mind (Thorvaldsen, Davidsen, Bronnum‐Hansen, & Schroll, 1999), the secondary prevention after stroke seems more important than ever. In 1998, the Copenhagen Stroke Study reported a recurrence rate of 23% (Jorgensen, Nakayama, Reith, Raaschou, & Olsen, 1997). According to the Danish Stroke Registry, our national recurrence rate was 25% in 2011 (Danish Stroke Registry, 2011).

Hypertension is an important risk factor for stroke recurrence (Friday, Alter, & Lai, 2002; Jorgensen et al., 1997; Lai, Alter, Friday, & Sobel, 1994; Prencipe et al., 1998). Lowering blood pressure (BP) after stroke or transitory ischemic attack (TIA) by 10/5 mm Hg has been associated with reduced risk of stroke recurrence by 24% and myocardial infarction (MI) by 21% ( Rashid, Leonardi‐Bee, & Bath, 2003).

Observational studies have demonstrated the difficulties in lowering BP after stroke with rates of BP treated to target ranging from 28% to 73% (Girot et al., 2005; Hornnes, Larsen, & Boysen, 2010; Johnson, Rosewell, & James, 2007; Paul & Thrift, 2006) and interventions aimed at control of BP after stroke have not yet found a successful model (Adie & James, 2010; Chiu et al., 2008; Ellis, Rodger, McAlpine, & Langhorne, 2005; Hornnes, Larsen, & Boysen, 2011; Johnston et al., 2010; Joubert et al., 2009). Fahey and coworkers have reviewed the literature aimed at improving control of BP in hypertensive subjects. One large study using an organized system of regular visits to a clinic was efficient in producing a large decrease in BP and reduction of all‐cause mortality compared to referral to usual primary care. This was achieved by using a stepwise escalation of treatment until target was reached (Fahey, Schroeder, & Ebrahim, 2006). Other methods had variable or no effect, only nurse‐ or pharmacist‐led care seemed promising.

### 1.1 Aims and hypotheses

The aim of the present study was to test the hypotheses that follow‐up after stroke in a specialized nurse‐led physician supervised clinic with stepwise escalation of BP‐ and lipid‐lowering treatment would result in

Primary endpoint:

A greater proportion of participants with BP at target.

Secondary endpoints:

A greater reduction of BP.

A greater proportion of participants with LDL‐cholesterol treated to target.

A greater reduction of LDL‐cholesterol.

Longer time to recurrence of stroke, MI, and death.

In the intervention group compared to controls.

## 2 MATERIALS AND METHODS

Before the initiation of the study, the authors attended a 3‐day course in treatment of hypertension arranged by the Danish Society of Hypertension. The recommendations of our national guidelines regarding BP targets were in line with those given by the American Stroke Association in force at the time of initiation of the study: “An absolute target BP level and reduction are uncertain and should be individualized.” (Furie, Kasner, & Adams, 2011). Following the advice given by the Danish Society of Hypertension, we used the following targets: A BP <140/90 mm Hg was considered at target in nondiabetic patients. In patients aged 80 years or more, a BP of 150/90 mm Hg was acceptable if further treatment was not tolerated. In case of severe carotid stenosis or a history of ischemic heart disease, BP should not be lower than 130/80 mm Hg. In patients with diabetes or hemorrhagic stroke, we aimed at a BP <130/80 mm Hg. Untreated patients without hypertension were categorized as normotensive, untreated hypertensive patients as having unknown hypertension, treated patients without hypertension as treated to target, and treated patients with hypertension as having untreated hypertension.

LDL‐cholesterol should be < 2.5 mmol/L in patients with IS or TIA in nondiabetic patients and in case of diabetes <2.0 mmol/L.

A sample size calculation showed that 24 patients in each group were needed to show a difference of 10 mm Hg in the development of systolic BP (80% power).

### 2.1 Study sample and setting

From June 2012 to February 2013, all patients diagnosed with a stroke or TIA at the stroke unit of Herlev Gentofte Hospital, University of Copenhagen were considered for inclusion in the study. Patients should be without cognitive deficits that would prevent their active participation and they should be discharged to their own home. The last author used computer‐generated block randomization procedures with stratification by hypertension (1:1). The allocation sequence was concealed, and we aimed at equal numbers in the two groups. Shortly before discharge, the first author approached eligible patients for oral and written information about the study. Where written informed consent to participation was achieved, BP was measured before a concealed envelope administered by a secretary was opened revealing the allocation to either intervention or control group.

The research protocol was approved by the ethics committee of the Capital Region of Denmark (H‐3‐2011‐152) and by the Danish Data Protection Agency (2012‐41‐0429). The study was conducted according to all common ethical standards including the rules given by the Declaration of Helsinki. Patients randomized to the control group had the usual treatment: one visit in the outpatient clinic of the stroke unit 3 months after discharge. Patients randomized to the intervention group had an appointment with the first author within 1 week after discharge. The first author undertook all visits in the preventive clinic.

### 2.2 Procedures and intervention

Blood pressure was measured at every visit after at least 5 min rest in a sitting position in an armchair. BP was measured simultaneously in both arms followed by two measurements with 10‐min intervals using the arm with the highest systolic BP. In case of hypertension, the first author would suggest initiation or intensification of antihypertensive treatment. The last author would accept or suggest an alternative and do the prescription. Patients would come to the clinic for control of BP and relevant blood tests every 3–4 weeks until BP was at target. After 5 weeks on lipid‐lowering drugs, treatment was intensified if needed. Patients who did not tolerate lipid‐lowering medication were referred to a dietitian. In motivated patients, home BP measurements were performed using patients' own monitor or by lending patients a BP monitor between visits.

Patients in the intervention group had a mean of five visits to the clinic with addition of new drugs rather than adding more of the same drug in case of hypertension. Although we used minimum doses to prevent adverse effects, many patients had unacceptable side effects necessitating change to another class of antihypertensive drug.

Patients were informed about the importance of lifelong adherence with all preventive medication. Those with elevated BP or receiving antihypertensive treatment were advised in salt reduction, smokers were advised to stop smoking, and all patients were informed about the benefits of 30 min of moderate physical activity daily. Likewise, information about the risk of an intake of alcohol above seven drinks per week in women and fourteen drinks in men was part of the program as well as the benefits of weight reduction in overweight patients with hypertension or diabetes.

### 2.3 Follow‐up

Participants in both groups were invited to the usual follow‐up visit 3 months after discharge at the outpatient clinic of the stroke unit as well as a follow‐up visit in the study a median of 9 (IQR 8–11) months after inclusion.

In accordance with the protocol, the final follow‐up visits were performed by nurses in the outpatient clinic with measurement of BP and blood cholesterols. Patients were asked not to reveal their group allocation but blinding of the nurses was not possible. Patients were interviewed about adherence to all preventive medications as well as their present lifestyle. For practical purposes, a minority of visits were performed by the first author. To do intention to treat analyses, we used last observation carried forward regarding the endpoints of the study where patients had died or did not respond to the invitation to a follow‐up visit. Thus, we used the last recorded values in five patients in the intervention group and in seven controls.

After a median of 65 months (IQR: 61–66) from inclusion, data on vascular events and death were attained from the hospital‐based medical records covering all hospitals of the region.

### 2.4 Statistics

Data were entered into Excel and imported into SAS. Statistical analyses were performed by the first author according to a pre‐established statistical analysis plan. We used chi‐square test (for the primary outcome) or Fisher's exact test as appropriate for comparison of proportions, and for change from baseline, we used McNemar's test. For continuous variables, we used *t* test or Mann–Whitney's test*. Change from baseline was analyzed by the paired *t* test or Wilcoxon signed rank sum test* (*where data were not normally distributed). We used SAS 9.4 for Windows, and *p* < .05 was considered significant.

## 3 RESULTS

**FIGURE 1:**
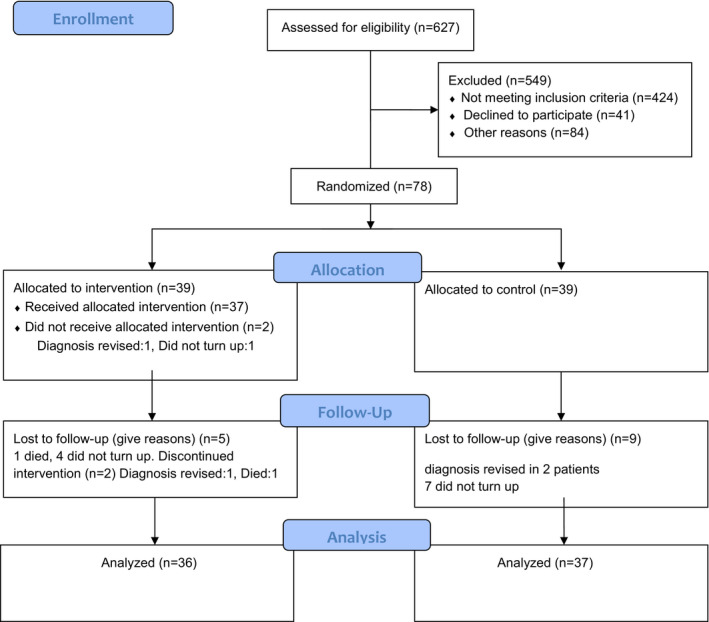
Flowchart of participants

We included 78 patients in the study. Due to revision of stroke diagnoses in four participants and as one participant never turned up for the intervention, data on 73 participants were available for follow‐up (Figure 1). The median stay in hospital was 4 days (IQR: 3–6). As seen from Table 1, most participants had no or slight disability.

**TABLE 1:** Baseline characteristics of 73 patients

Characteristics	All (*n* = 73)	Intervention (*n* = 36)	Control (*n* = 37)	*p*
Sex, female	29 (40)	15 (42)	14 (38)	.74
Age (years), mean ± *SD*	66 ± 12	63 ± 13	68 ± 11	.08
Length of education				
<10 years	12 (17)	6 (17)	6 (16)	.95
10–12 years	22 (30)	10 (29)	12 (33)	
>12 years	38 (53)	19 (54)	19 (51)	
Diagnosis of stroke				
Ischemic stroke	63 (87)	33 (92)	30 (81)	.60^a^
TIA	9 (12)	3 (8)	6 (16)	
Hemorrhagic stroke	1 (1)		1 (3)	
Recurrent stroke	11 (15)	5 (14)	6 (16)	1.00^a^
Modified Rankin Scale score >2	5 (6)	1 (3)	4 (11)	.36^a^
Antihypertensive medication before stroke	39 (53)	15 (42)	24 (65)	.047
Antihypertensive medication at discharge	46 (63)	20 (56)	26 (70)	.19
Cholesterol‐lowering medication before stroke	25 (34)	11 (31)	14 (38)	.51
Cholesterol‐lowering medication at discharge	65 (89)	35 (97)	30 (81)	.03
Diabetes at baseline	14 (19)	5 (14)	9 (24)	.37^a^
Diabetes at discharge	16 (22)	6 (17)	10 (27)	.29
Atrial fibrillation at baseline	7 (10)	3 (8)	4 (11)	1.00^a^
Atrial fibrillation at discharge	12 (16)	6 (16)	6 (17)	1.00
Unhealthy dieting^b^	59 (82)	25 (71)	34 (92)	.03
Current smoking	19 (26)	11 (31)	8 (22)	.62
Alcohol above limits^c^	23 (32)	12 (34)	11 (30)	.68
Sedentary lifestyle^d^	17 (24)	9 (26)	8 (22)	.68
BMI ≥25	46 (63)	24 (67)	22 (59)	.52
Self‐rated health: fair, poor, or very poor	34 (47)	15 (43)	19 (51)	.47

Note

Values are expressed as frequencies (%) or as mean ± standard deviations.

^a^ Fisher's exact test.

^b^ Less than 600 g of fruit and vegetables per day, fish for dinner less than twice per week.

^c^ More than seven drinks per week in women/more than 14 drinks per week in men.

^d^ Less than 30 min of moderate physical activity per day.

**FIGURE 2:**
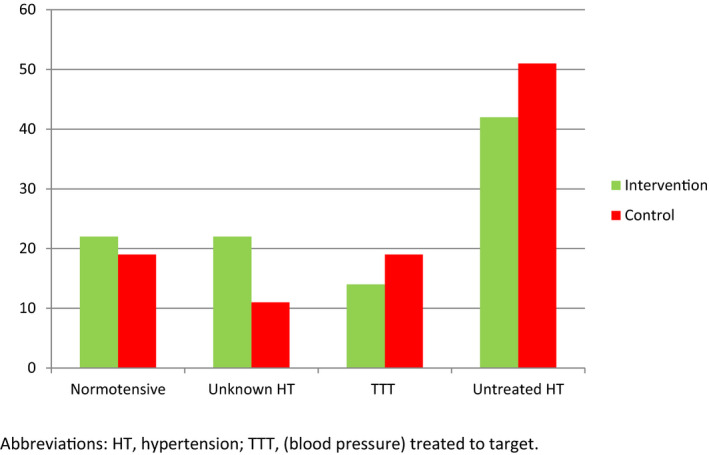
Blood pressure and treatment of hypertension at baseline in 73 patients (%)

Less than 20% of patients had a baseline BP treated to target (Figure 2). Twenty‐eight patients (78%) in the intervention group and 29 patients (78%) in the control group had a 3‐month visit in the outpatient clinic. Here, 15 patients (42%) in the intervention group had their BP and blood cholesterol measured and so had 23 patients (62%) in the control group. At follow‐up, patients in both groups reported a median of two visits including BP measurement at the general practitioner's office since discharge from hospital.

### 3.1 Primary endpoint

**FIGURE 3:**
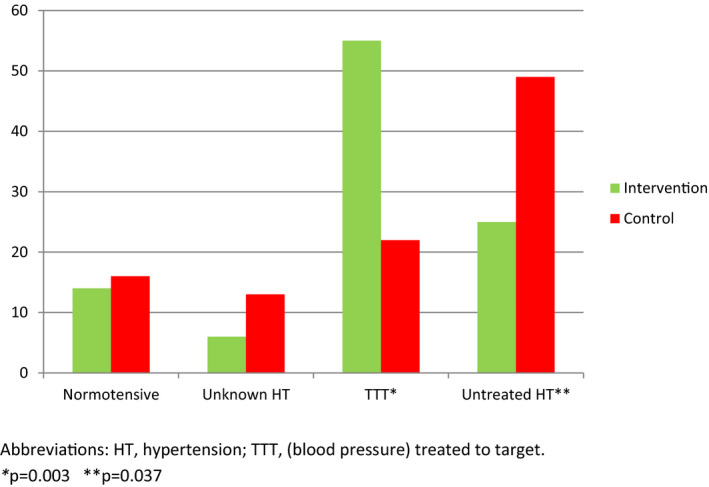
Blood pressure and treatment of hypertension at follow‐up in 73 patients (%)

Follow‐up visits showed that 25 patients (69%) in the intervention group had a BP at target versus 14 (38%) of controls (*p* = .007). In four patients (10%) in the intervention group, antihypertensive medication remained unchanged since discharge versus 23 (62%) of controls (*p* < .0001) illustrated by the differences in BP treated to target as well as untreated hypertension in Figure 3.

### 3.2 Secondary endpoints

Median reduction in systolic BP was 11 mm Hg (−5–19) with 14 mm Hg (IQR: 5–21) in the intervention group and 3 mm Hg (IQR −11–17) in the control group (*p* = .045). Median reduction in diastolic BP was 2 mm Hg (−2–11) with 7 mm Hg (IQR −1–13) in the intervention group and 1 mm Hg (IQR −6–8) in the control group (*p* = .04).

There was no difference between the groups regarding LDL‐cholesterol treated to target with 32 patients (89%) at target in the intervention group versus 29 patients (78%) in the control group (*p* = .21). We found significant reductions in LDL‐cholesterol in both groups, but no difference between the groups: 1.6 (IQR: 0.4–2.2) mmol/L in the intervention group versus 0.8 (IQR: 0.4–1.8) mmol/L among controls (*p* = .18).

In 11 patients (31%) in the intervention group, cholesterol‐lowering medication remained unchanged since discharge versus 29 (78%) of controls (*p* < .0001).

The combined endpoint of both BP and LDL‐cholesterol at target was achieved in 22 (61%) of patients in the intervention group and in 10 patients (27%) in the control group (*p* = .003). At the end of the study, 24 patients (68%) in the intervention group measured their BP at home versus 14 (38%) of controls (*p* = .03).

The only significant change in lifestyle was a reduction in current smokers by four in the control group (Table 2).

**TABLE 2:** Nine‐month follow‐up of 73 patients

Characteristics	All (*n* = 73)	Intervention (*n* = 36)	Control (*n* = 37)	*p*
Systolic BP, mm Hg, mean ± *SD*	134 ± 21	130 ± 17	137 ± 24	.12
Diastolic BP, mm Hg, mean ± *SD*	78 ± 11	78 ± 10	78 ± 12.8)	.94
Antihypertensive medication	55 (75)	29 (81)	26 (70)	.31
100% compliance with AHM (*n* = 45)	38 (84)	23 (89)	15 (79)	.38
LDL‐cholesterol, mmol/L (*n* = 72), mean ± *SD*	1.9 ± 0.8	1.9 ± 0.7	2.0 ± 0.8	.66
Cholesterol‐lowering medication	64 (88)	32 (89)	32 (86)	.76
100% compliance with CLM (*n* = 52)	46 (89)	24 (86)	22 (92)	.50
Unhealthy dieting^b^	59 (81)	26 (72)	33 (89)	.76
Current smoker	15 (21)	11 (31)	4 (11)	.046^a^
Alcohol >limits^c^	20 (27)	11 (31)	9 (24)	.55
Sedentary lifestyle^d^	16 (22)	7 (20)	9 (24)	.66
BMI ≥25	43 (59)	21 (58)	22 (60)	.92

Note

Values are expressed as frequencies (%) or as mean ± standard deviations.

Abbreviations: AHM, antihypertensive medication; CLM, cholesterol‐lowering medication.

^a^ Fisher's exact test.

^b^ Less than 600 g of fruit and vegetables per day, fish for dinner less than twice per week.

^c^ More than seven drinks per week in women/more than 14 drinks per week in men.

^d^ Less than 30 min of moderate physical activity per day.

Regarding vascular complications and death, we found 32 events in 22 patients after a median of 65 months. Median time to first event was 26 months (IQR: 4–49) with a median of 44 months (IQR: 11–49) in the intervention group and 19 months (IQR: 4–37) in the control group (*p* = .32). All in all, we found 11 events in nine patients in the intervention group: two recurrent strokes, three cases of TIA, and six patients died versus 21 events in 13 patients in the control group: seven recurrent strokes, five cases of TIA, one MI, and seven patients died (*p* = .49).

## 4 DISCUSSION

In this randomized clinical trial, a larger proportion of patients in the intervention group compared to controls had BP within the above‐mentioned limits and the study fulfilled the aim of the primary endpoint.

A systematic review of interventions aimed at modifiable risk factor control for secondary prevention of stroke revealed improvement in achieving BP target (Bridgwood et al., 2018). However, as opposed to our study, the review showed no significant change in systolic or diastolic BP.

In a study of integrated care with five prearranged visits to patients' general practitioner versus usual care, systolic BP at target set to 140 mm Hg was found in 75% versus 58% at 12‐month follow‐up (Joubert et al., 2009). We set individual targets for BP according to patients' type of stroke, comorbidities, and age. This is well in line with recommendations given by European Society of Hypertension (Mancia et al., 2013), but as stated by Boan, Lackland, and Ovbiagele (2014), not quite in accordance with international stroke guidelines.

In a study, where patients with minor stroke were randomized to six clinic visits by a pharmacist (intervention) or by a nurse (active control) aiming at treating both BP and LDL‐cholesterol to target, 43% of patients in the pharmacist‐led clinic met those two targets and so did 31% in the nurse‐led clinic (McAlister et al., 2014). In our study, this combined endpoint was met in 22 (61%) of patients in the intervention group. Regarding BP in control, 80% of patients in the pharmacist‐led clinic had systolic BP in control after 6 months versus 90% in the nurse‐led clinic. This is a far greater proportion than the 69% in our study. However, almost two‐thirds of patients had a baseline BP within the limits. The opposite was the case in our study with two‐thirds presenting with elevated BP. Both studies show that a dedicated follow‐up with stepwise escalation of preventive medication may be the way to reach the targets of the two important risk factors for recurrent stroke. In both studies, five‐six visits were needed, which is far beyond our usual treatment. However, despite visits to the outpatient clinic as well as to the general practitioner, the proportion of patients with unchanged medication since discharge in the control group illustrate the necessity of frequent visits to a dedicated preventive facility. Considering the preventive effect of BP lowering, and—though insignificant—the difference in time to first event as well as the smaller proportion of events in the intervention group as found in our study, it may be well worth the time and resources for patients, their relatives, and society.

### 4.1 Strengths and limitations

Our study has some limitations. Most participants had a minor stroke and patients had to be independent and without severe cognitive deficits, which is not representative of a general stroke population. With only 73 participants, caution is called for in the drawing of conclusions from the results. Nonetheless, we decided to reorganize the outpatient clinic of our stroke unit as of October 2014 implementing strategies of the present study.

The strength of the study is the individual target for BP taking into account the diagnosis of stroke as well as important comorbidity as recommended by Boan et al. (2014) Five‐year follow‐up on vascular complications and death is another important advantage.

## 5 CONCLUSIONS

In conclusion, the feasibility study has demonstrated that timely follow‐up of stroke patients in a dedicated preventive outpatient clinic may result in BP and cholesterol treated to target in most patients. To some extent, it may postpone time to stroke recurrence, MI, and death.

## CONFLICT OF INTEREST

None.

## AUTHOR CONTRIBUTIONS

Both authors have made substantial contributions to conception and design and acquisitions and analyses and interpretation of data and have been involved in writing the manuscript and given final approval of the version to be published. Both authors have participated sufficiently in the work to take public responsibility for the content and agree to be accountable for all aspects of work in ensuring that questions related to the accuracy or integrity of any part of the work are appropriately investigated and resolved.

## Data Availability

The data that support the findings of this study are not available due to national privacy or ethical restrictions.
